# Absolute Distance Measurement Using Frequency-Scanning Interferometry Based on Hilbert Phase Subdivision

**DOI:** 10.3390/s19235132

**Published:** 2019-11-23

**Authors:** Shuo Jiang, Bo Liu, Huachuang Wang, Bin Zhao

**Affiliations:** 1 University of Chinese Academy of Sciences, Beijing 100049, China; jingjingyua@163.com; 2 Institute of Optics and Electronics, Chinese Academy of Sciences, Chengdu 610209, China; wanghuachuang@163.com (H.W.); zhaobin@ioe.ac.cn (B.Z.)

**Keywords:** frequency-scanning interferometry, distance measurement, Hilbert phase subdivision

## Abstract

In order to eliminate the influence of laser frequency nonlinearity, the frequency-scanning interferometry (FSI) often uses the beat signal of an auxiliary interferometer as the external clock. The time points at every amplitude peaks and bottoms of the auxiliary beat signal are selected as the sampling time points for the main interferometer signal. To satisfy the Nyquist sampling requirement, the optical path difference (OPD) of the delay fiber in auxiliary interferometer should be at least twice longer than the measurement distance. In this paper, we proposed a method to shorten the length of delay fiber. The Hilbert transform was used to extract the phase of the auxiliary interference signal and calculate the time points corresponding to subdivided phase intervals. Then, the main interference signal was resampled at these moments, and the fast Fourier transform was performed on the resampled signal. The experimental results showed that the target at the distance of about 5 m was measured when the OPD of the auxiliary interferometer was about 4.5 m. The standard deviation of the distance measurement results could reach 4.64 μm.

## 1. Introduction

Frequency modulation first came out in the radio-frequency field to measure the distance of target in free-space, then it was introduced into the field of optics in recent years [1,2]. The frequency-scanning interferometry (FSI) laser ranging is a well-known absolute distance measurement method with high precision and resolution. FSI uses the linear characteristic of the laser frequency to calculate the distance information by measuring the beat frequency of the interference signal [3,4,5]. Because the frequency of the laser is much higher than radio-frequency, a wide modulation bandwidth can be easily achieved by laser-scanning technology. With the development of an external cavity diode laser (ECDL), the modulation bandwidth can easily reach several THz, which helps to improve the distance resolution to tens of μm. FSI laser ranging technology has the advantages of non-contact measurement and no dead measurement zone; furthermore, it does not require a cooperative target. Therefore, it plays an irreplaceable role in aerospace, machining, and other high precision metrology fields [6].

In coherent detection, the optical frequency nonlinearity in the process of laser modulation leads to the frequency spectrum of beat signal broadening, resulting in a decrease in spatial resolution. The nonlinearity is caused by the low control precision of the tuning motor, hysteresis, and the creep of the piezoelectric actuator. In order to solve this problem, several methods have been developed. One way is to correct the scanning optical frequency to be linear through a feedback system directly. In 2009, Satyan demonstrated a wideband frequency sweeps using a semiconductor laser in an optoelectronic feedback loop. With a reference signal, a transform-limited linear sweep of 100 GHz in 1 ms was achieved, and a spatial resolution of 1.5 mm was demonstrated [7]. In the same year, Roos et al. used an external cavity semiconductor laser with two phase-locked loops to realize a precise linearization of laser frequency, and the bandwidth reached up to 4.8 THz. Moreover, the measurement accuracy of 86 nm for a target at a distance of 1.5 m was achieved [8]. In 2011, Liyama et al. used a single-mode vertical-cavity surface-emitting laser as the light source. It swept the laser frequency by injection current method, and the spatial resolution of 250 μm in the air was achieved for a target at about 4 cm [9]. Furthermore, the method based on photoelectric feedback to eliminate optical frequency nonlinearity is complicated and costly [10]. In addition to directly improving the laser source, measuring the phase difference of the interference signal is another method to eliminate the nonlinearity of frequency modulation. In 2018, Deng proposed a ranging method that used the Hilbert–Huang transform to extract the phase of the interference signal, effectively solving the laser sweep nonlinearity and improving the accuracy of the distance measurement. The experiment proved that the standard deviation of this FSI ranging system was only 0.7 μm [10]. Also, it is an ideal method to eliminate frequency modulation nonlinearity by resampling with an auxiliary interferometer. In 2014, Shi used an external cavity semiconductor laser and an auxiliary interferometer to resample the measurement signal at every peak and bottom time of the reference interferometer signal. The experiments demonstrated a 50 μm range resolution at 8.7 m with a delay fiber of 30 m [11]. In 2018, Lu proposed a frequency scanning interferometry absolute distance measurement to measure a non-cooperative target located at a distance of 10 m with a delay fiber of 200 m and achieved a standard deviation (STD) of 3.43 μm [12]. 

In the FSI laser ranging system, the fiber Mach–Zehnder (M–Z) interferometer is usually used as an auxiliary interferometer to eliminate the influence of frequency modulation nonlinearity. The beat signal of the auxiliary interferometer is regarded as an external clock. The Nyquist sampling theorem requires that the optical path difference of the auxiliary interferometer should be at least twice longer than the measurement distance. For a distance of tens of meters, a longer delay fiber is required, which will increase the size and weight of the system. In order to reduce the length of delay fiber, we proposed a method based on the Hilbert phase subdivision. Through the Hilbert transform, the phase of the auxiliary interference signal was accurately extracted. With the Hilbert phase spectrum, the time points corresponding to the subdivision phase intervals could be obtained, and the main interference signal was sampled at these time points. In this way, the length of delay fiber in auxiliary interferometer could be shortened. This method is suitable for large-scale free space distance measurements and could be used in industrial applications.

## 2. Materials and Methods

### 2.1. The Basic Principle of FSI

**Figure 1:**
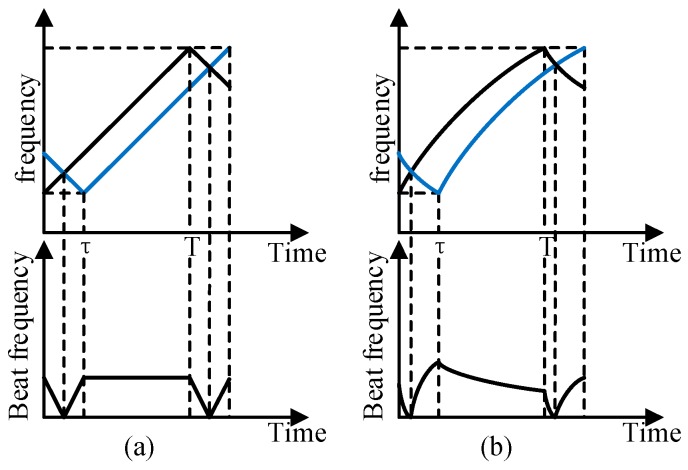
The waveform of the FSI (frequency-scanning interferometry) ranging system: (**a**) The beat signal for laser linear scanning; (**b**) The beat signal for laser nonlinear scanning.

Figure 1 illustrates the basic measurement principle of FSI laser rangefinder. The black line represents the local oscillator, and the blue line represents the echo optic signal from the target; the beat frequency of them could be expressed as:

(1) f=k(t)⋅τ

where k(t) is the instantaneous scanning speed of the laser, and τ is the time delay between the local oscillator and echo.

Figure 1a shows the condition that the optical frequency is linearly modulated, where k(t) should be a constant value k. Thus, the beat frequency is proportional to the time delay τ, and the distance can be written as:

(2) $$R=\frac{\tau \cdot c}{2}=\frac{f\cdot c}{2\cdot k}$$

where c is the speed of light in vacuum.

Since actual lasers usually cannot achieve ideal linear modulation directly while maintaining wide bandwidth, there is nonlinearity, as shown in Figure 1b, so the frequency of the beat signal is no longer fixed. Performing fast Fourier transform on the interference signal directly would reduce the accuracy of range.

### 2.2. System Description

**Figure 2:**
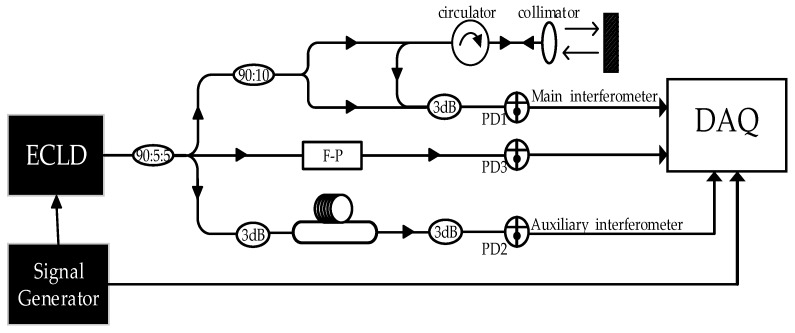
Schematic of an FSIlaser ranging system.

The schematic diagram of our system is shown in Figure 2. The system consisted of an external cavity diode laser (ECDL), two optical fiber M–Z interferometers, one of which was an auxiliary interferometer, and the other was the main interferometer for the distance measurement. 

In this system, the ECDL was the laser source of the whole system. The signal generator provided a trigger signal that enabled the laser source and the data acquisition card (DAQ) to start working at the same time. While the laser frequency was being swept, the light was divided into three paths through a coupler. The uppermost beam entered the main interferometer and was split into two parts, one as the local oscillator, and the other was transmitted to the target. The reflected light interfered with the local oscillator on the photodetector 1 and obtained the main interference signal. The bottom beam entered the auxiliary interferometer and was split into two arms, one as the local oscillator, the other was injected into the delay fiber, and the auxiliary interference signal was obtained by the photodetector 2. The middle beam was transmitted into the Fabry–Pérot (F–P) cavity, generating the transmission resonance signal on the photodetector 3.

### 2.3. The Principle of the Frequency-Sampling Method

The electric field of the scanning optic can be written as:

(3) $$E(t)=E_{0}\cos(2\pi f_{0}\cdot t+\pi \cdot k(t)\cdot t^{2})$$

where $E_{0}$ is the amplitude of the signal, $f_{0}$ is the initial frequency of the laser.

The laser in the two arms of the interferometer can be expressed as E(t) and E(t+τ), and the interference signal is:

(4) $$\begin{array}{l} I(t)={\left|E(t)+E(t+\tau )\right|}^{2}\\ ={E_{0}}^{2}(t)+{E_{0}}^{2}(t+\tau )+2{E_{0}}^{2}\cos[\varphi (t+\tau )-\varphi (t)]\\ =E^{2}(t)+E^{2}(t+\tau )+2{E_{0}}^{2}\cos\{2\pi \tau [f_{0}+k(t)t]+\pi k(t)\tau^{2}\} \end{array}$$

where φ(t) and φ(t+τ) represent the instantaneous phase of E(t) and E(t+τ), respectively. Since the first two terms are constant, and $\tau^{2}$ is very small as the measurement distance of the system is under a few tens of meters, so they could be ignored, and the interference signal is:

(5) $$f(t)=f_{0}+k(t)\cdot t$$

(6) $$I(t)=I_{0}\cos[2\pi f(t)\tau ]$$

where $I_{0}$ represents the amplitude of the interference signal, and f(t) represents the instantaneous frequency of the laser. From Equation (6), we can see that the phases of interference signals with the same laser source are proportional to time delay.

The expression of the interference signal of the auxiliary interferometer is:

(7) $$I_{A\mathrm{ux}}(t)=I_{A\mathrm{ux}}\cos[2\pi f(t)\tau_{0}]$$

where $I_{Aux}$ and $\tau_{0}$ are the amplitude and the time difference, respectively. The common method is to sample the main interferometer signal at the extreme positions of $I_{Aux}(t)$ with the equal phase interval of π. The resampling signal can be obtained as follows:

(8) $$I_{m}(n)=I_{m}\cos(2\pi \frac{n}{2\tau_{0}}\tau_{m})$$

where $I_{m}$ is the amplitude of the main interference signal, $\tau_{m}$ is the time delay, and n is the number of resampling points. It can be seen from the Equation (8) that the nonlinearity of the optical frequency in the interference signal is eliminated.

### 2.4. The Principle of Hilbert Phase Subdivision Resampling

According to the Nyquist sampling theorem, only when the maximum frequency of the sampled signal is less than half of the sampling frequency, the information of the original signal could be recovered. Therefore, the optical path difference of the auxiliary interferometer should be at least twice longer than the measured distance. In order to reduce the requirement for the length of the delay fiber in an auxiliary interferometer, the Hilbert transform is adopted to accurately extract the phase of the auxiliary interference signal. The auxiliary interferometer delay fiber is reduced by obtaining a smaller phase of the auxiliary interference signal to increase the frequency of the resampling.

For the auxiliary interference signal, its Hilbert transformation is:

(9) $$I_{hil}(t)=Hilbert[I_{Aux}(t)]=\frac{1}{\pi }\int_{-\infty }^{\infty }\frac{I_{Aux}(t)}{t-\tau }d\tau$$

And the analytic expression of the signal can be expressed as:

(10) $$I(t)=I_{Aux}(t)+j\cdot I_{hil}(t)=A(t)\cdot e^{j\cdot \theta (t)}$$

where A(t) represents the amplitude of the analytic signal, θ(t) represents the instantaneous phase, and they can be calculated as:

(11) $$A(t)=\sqrt{{I^{2}}_{Aux}(t)+{I^{2}}_{hil}(t)}$$

(12) $$\theta (t)=\arctan\frac{I_{hil}(t)}{I_{Aux}(t)}$$

**Figure 3:**
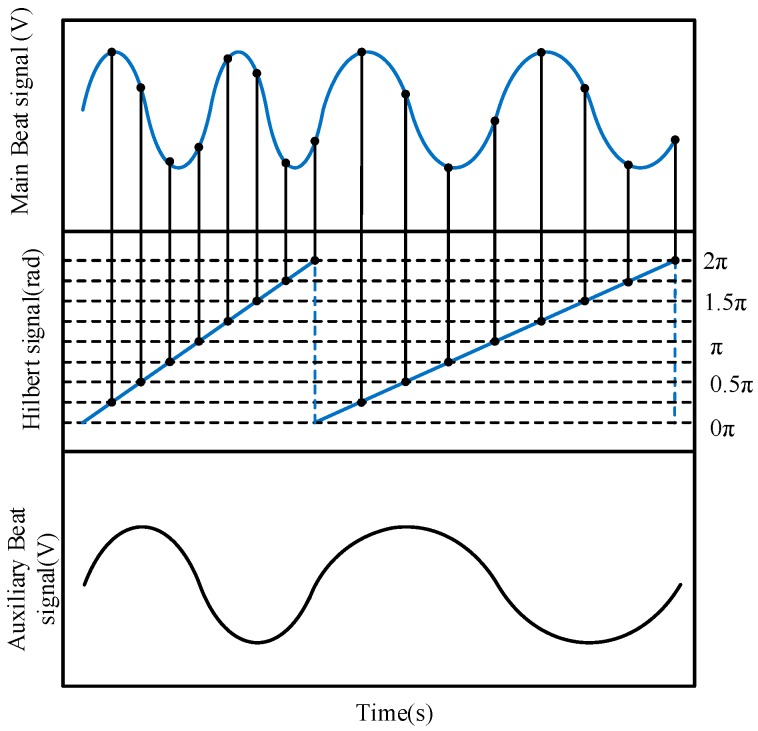
Schematic diagram of resampling based on Hilbert phase subdivision.

Through the Hilbert transform, we could obtain the instantaneous phase of the auxiliary beat signal. In order to obtain smaller equal phase intervals, the phase period 2π can be divided into α equal parts appropriately. Finding the time points corresponding to these phases, and getting the resampling points from the main beat signal, the resampled signal is gained. Figure 3 is the schematic diagram of the process when α = 8. The resampled signal can be written as:

(13) $$I_{m}(n)=I_{m}\cos(2\pi \frac{n}{\alpha \cdot \tau_{0}}\tau_{m})$$

As long as $\frac{2\tau m}{\alpha }<\tau_{0}$, the sampling theorem can be met to recover the frequency information of the resampled signal.

## 3. Experiments and Results

### 3.1. Experiment

The experimental structure diagram of the FSI ranging system has been shown in Figure 2. The system was arranged on the air floating optical experimental platform to isolate vibration influence. An external cavity diode laser (New Focus TLB-6728, Irvine, CA USA) acted as the laser source of the system, with a line width of 200 kHz. The sweep range was from 1545 nm to 1555 nm, and the tuning speed was 5 nm/s. The system also included an optical fiber auxiliary interferometer, the main interferometer, and an F–P cavity (Thorlabs SA200-12B, Newton, MA USA). The delay fiber of the auxiliary interferometer was bundled neatly on a metal cylinder and fixed. The optical path difference (OPD) of the fiber was 4519.375 mm, which was calibrated by an optical frequency domain reflectometer (Luna OBR 4600, Blacksburg, the US). The signal generator generated a pulse signal with a period of 3 s, which was used as the synchronous trigger signal of the laser and data acquisition card (DAQ) (Spectrum M4i.4421-x8, Grosshansdorf, Germany). The sampling frequency set by the acquisition card was 500 kHz. While the laser frequency was being swept, 5% of the laser entered the auxiliary interferometer, 5% of the laser entered the F–P cavity, and the remaining 90% entered the main interferometer.

According to the number of resonance spectral peaks of the F–P cavity, the sweep range of the tuning laser was about 1.2 THz. The target was a corner reflector placed on a Hexapod Motion (Newport HXP-200, Irvine, CA USA), and the initial distance between the target and the FSI system was about 5 m. 

**Figure 4:**
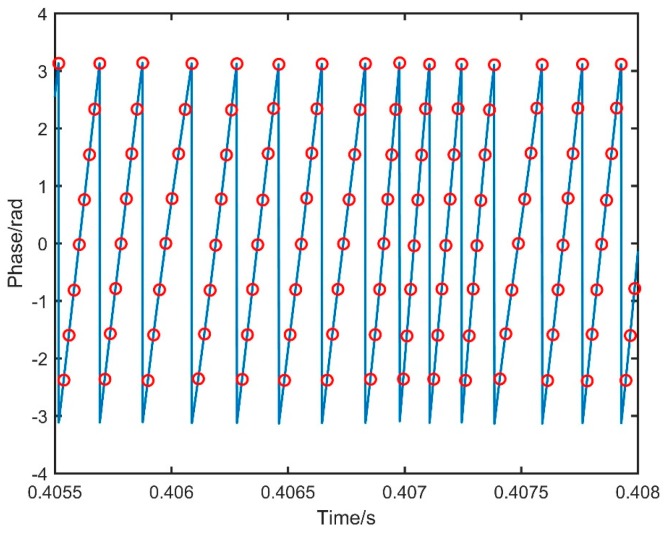
Hilbert transform of the auxiliary interference signal.

Parts of the auxiliary interference signal from 0.4055 s to 0.408 s is shown in Figure 4, and the phase of the auxiliary interference signal acquired by the DAQ could be obtained by performing Hilbert transform to it. For the maximum measurement, the distance of the system was set to 9 m, and $\frac{\pi }{4}$ was taken as the phase interval of resampling. In Figure 4, the red circles represent the positions of the corresponding phases. Therefore, the resampling time points could be obtained. Comparing with the former part from 0.4055 s to 0.407 s, the time points of the following part from 0.407 s to 0.4075 s were more compact, which demonstrated the nonlinearity of the laser scanning.

### 3.2. Results

**Figure 5:**
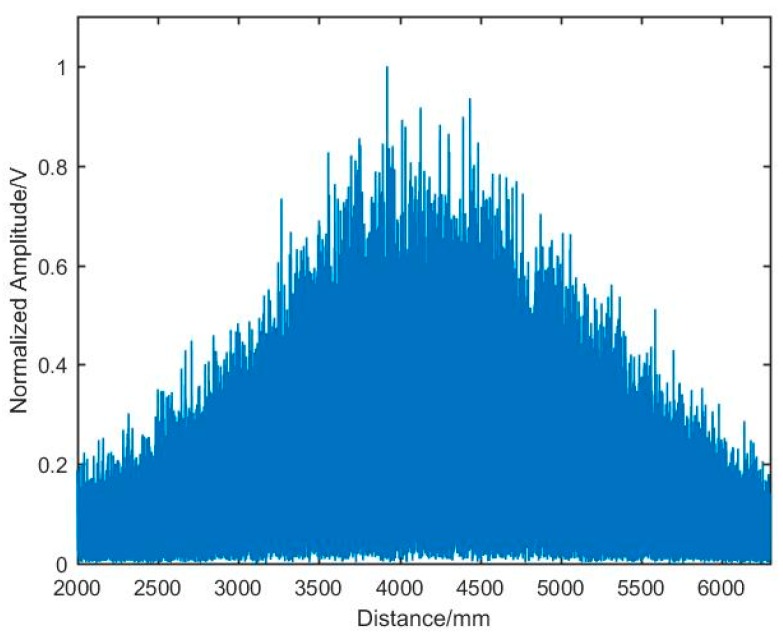
The distance spectrum without resampling.

Figure 5 shows the distance spectrum without the resampling method. FFT was used for the main beat signal directly, and then the distance spectrum was obtained, where a very severe broadening could be seen, and the target peak was submerged. 

**Figure 6:**
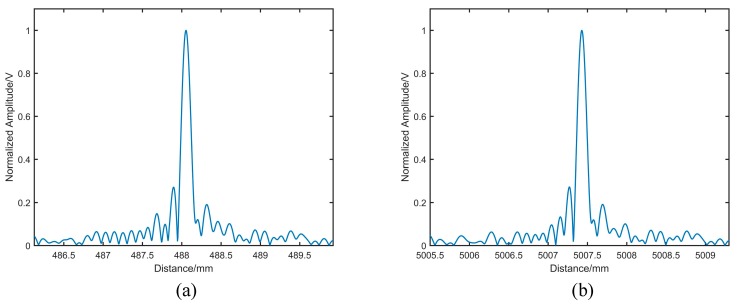
The results of the distance measurements: (**a**) The distance spectrum without the Hilbert phase subdivision resampling; (**b**) The distance spectrum with the Hilbert phase subdivision resampling.

Figure 6a shows the distance spectrum, resampling the main beat signal at every amplitude peaks and bottoms of the auxiliary beat signal. The distance spectrum became much narrower compared to that in Figure 5 for the nonlinearity been corrected. However, in Figure 6a, the peak of the spectrum was at around 488 mm, which was significantly different from the distance of the target. The reason for the phenomenon was that the frequency of the auxiliary beat signal was lower than the main beat signal, resulting in the Nyquist sampling theorem not being satisfied. Performing FFT on the resampled signal would cause spectral aliasing. Thus, the real distance of the target could not be accurately calculated.

Figure 6b shows the distance spectrum with the Hilbert subdivision resampling method. Reducing the interval of phase points from π to $\frac{\pi }{4}$ was equivalent to increasing the speed of resampling by 4 times. Therefore, the Nyquist sampling theorem was satisfied, and the peak of the spectrum was at a distance of approximately 5007 mm. 

**Figure 7:**
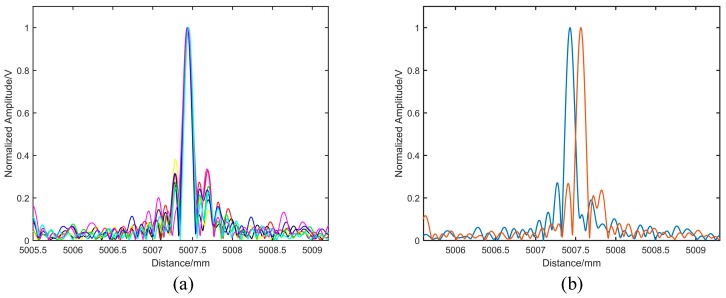
The distance measurement results: (**a**) Distance spectra for multiple measurements; (**b**) Result of the resolution experiment.

Figure 7a demonstrates the distance spectra of the target at the same position; the solid lines of different colors represent the results of multiple measurements. By calculation, the standard deviation of ten distance measurements was 4.64 μm. We moved the target backward by 135 μm and processed the two sets of data before and after the move. The separation of the distance spectra could be seen in Figure 7b, thus verifying the distance resolution of 135 μm, which was close to the theoretical calculation value of 125 μm. The reason for this was the dispersion in an auxiliary interferometer, making the time delay in Equation (7) to vary with the modulation bandwidth. Then, the resample signal did not strictly equal phase intervals, and the full width at half maximum (FWHM) of the spectrum was broadened. 

## 4. Discussion

The FSI absolute distance measurement method is high precision and high resolution ranging method. However, the nonlinearity of laser frequency modulation affects its accuracy and resolution. The method, using the extreme points of the auxiliary interference signal as the resampling time, requires the length of delay fiber in an auxiliary interferometer, at least, twice longer than the measured distance. Using the Hilbert transform to extract the phase of the auxiliary interference signal can obtain a smaller equal phase. Then, the requirement for the length of the delay fiber is reduced. The most important thing in the process of data processing is the extraction of equal phase points. Resampling requires the auxiliary interference signal to be sufficiently smooth for the Hilbert transform, so we fixed the fiber auxiliary interferometer neatly on a metal cylinder. The sampling frequency of the DAQ was set to 20~25 times of the highest frequency of the signal and combined with the interpolation algorithm to the auxiliary and main beat signal. Consequently, a very smooth interference signal was obtained, and the Hilbert transform could be directly performed.

## 5. Conclusions

In this article, we presented a method for phase subdivision using a Hilbert transform and then resampling. It eliminated the influence of the nonlinearity of the laser frequency modulation and reduced the requirement for the length of the auxiliary interferometer delay fiber. This method was suitable for measuring the target at tens of meters far from the FSI system, and it could help shorten the length of the delay fiber and reduce the size and weight of the whole system. With the experimental verification, we achieved a standard deviation of 4.64 μm with a delay fiber shorter than twice the measurement distance, and the resolution reached 135 μm.
